# 3-Benzamidomethyl-4-[(*E*)-2-chloro­benzyl­ideneamino]-1*H*-1,2,4-triazole-5(4*H*)-thione

**DOI:** 10.1107/S1600536808040361

**Published:** 2008-12-10

**Authors:** Hoong-Kun Fun, Samuel Robinson Jebas, Jyothi N. Rao, B. Kalluraya

**Affiliations:** a X-ray Crystallography Unit, School of Physics, Universiti Sains Malaysia, 11800 USM, Penang, Malaysia; b Department of Studies in Chemistry, Mangalore University, Mangalagangotri, Mangalore 574 199, India

## Abstract

In the title compound, C_17_H_14_ClN_5_OS, the dihedral angles formed by the two benzene rings with the triazole ring are 66.88 (3) and 19.16 (3)°, and the benzene rings are inclined to each other with a dihedral angle of 78.40 (3)°. Inter­molecular N—H⋯O hydrogen bonds link the mol­ecules into layers parallel to the (100) planes, and centrosymmetric π–π stacking inter­actions [centroid–centroid distance = 3.7717 (5) Å] are formed between benzene rings in neighbouring layers.

## Related literature

For pharmaceutical and other applications of triazole compounds, see: Almasirad *et al.* (2004 ▶); Al-Soud *et al.* (2003 ▶); Amir & Shikha (2004 ▶); Kalluraya *et al.* (1996 ▶); Kawashima *et al.* (1987 ▶).
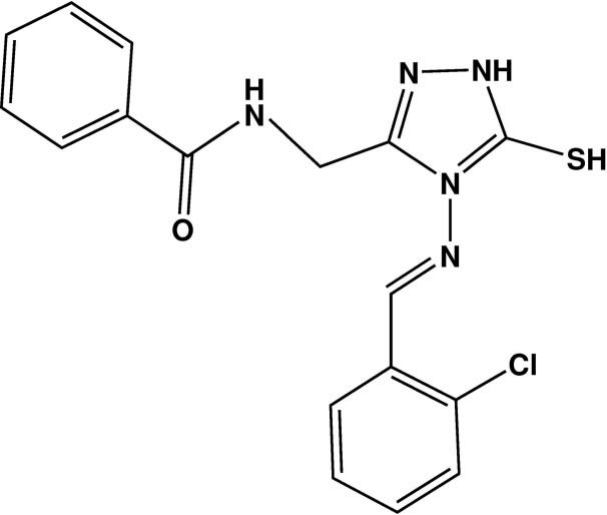


## Experimental

### 

#### Crystal data

C_17_H_14_ClN_5_OS
*M* *_r_* = 371.84
Monoclinic, 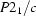

*a* = 17.0185 (6) Å
*b* = 8.0905 (3) Å
*c* = 12.8292 (5) Å
β = 105.962 (2)°
*V* = 1698.32 (11) Å^3^
*Z* = 4
Mo *K*α radiation
μ = 0.36 mm^−1^
*T* = 100.0 (1) K
0.70 × 0.48 × 0.15 mm

#### Data collection

Bruker SMART APEXII CCD diffractometer
Absorption correction: multi-scan (*SADABS*; Bruker, 2005 ▶) *T*_min_ = 0.785, *T*_max_ = 0.949
57891 measured reflections
7463 independent reflections
6349 reflections with *I* > 2σ(*I*)
*R*_int_ = 0.025

#### Refinement

*R*[*F* ^2^ > 2σ(*F* ^2^)] = 0.033
*wR*(*F* ^2^) = 0.098
*S* = 1.09
7463 reflections
234 parameters
2 restraints
H atoms treated by a mixture of independent and constrained refinement
Δρ_max_ = 0.59 e Å^−3^
Δρ_min_ = −0.23 e Å^−3^

### 

Data collection: *APEX2* (Bruker, 2005 ▶); cell refinement: *SAINT* (Bruker, 2005 ▶); data reduction: *SAINT*; program(s) used to solve structure: *SHELXS97* (Sheldrick, 2008 ▶); program(s) used to refine structure: *SHELXL97* (Sheldrick, 2008 ▶); molecular graphics: *SHELXTL* (Sheldrick, 2008 ▶); software used to prepare material for publication: *SHELXTL* and *PLATON* (Spek, 2003 ▶).

## Supplementary Material

Crystal structure: contains datablocks global, I. DOI: 10.1107/S1600536808040361/bi2320sup1.cif

Structure factors: contains datablocks I. DOI: 10.1107/S1600536808040361/bi2320Isup2.hkl

Additional supplementary materials:  crystallographic information; 3D view; checkCIF report

## Figures and Tables

**Table 1:** Hydrogen-bond geometry (Å, °)

*D*—H⋯*A*	*D*—H	H⋯*A*	*D*⋯*A*	*D*—H⋯*A*
N3—H1N3⋯O1^i^	0.85 (1)	1.89 (1)	2.7362 (10)	175 (1)
N1—H1N1⋯O1^ii^	0.84 (1)	2.29 (1)	2.9450 (10)	134 (2)

Symmetry codes: (i) 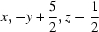; (ii) 
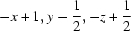
.

**Fig. 1.**
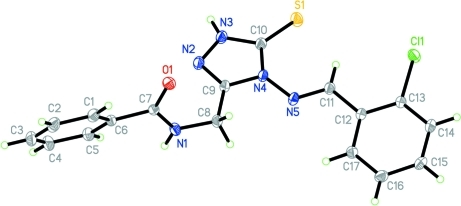
The molecular structure of the title compound, showing 50% probability displacement ellipsoids for non-H atoms.

**Fig. 2.**
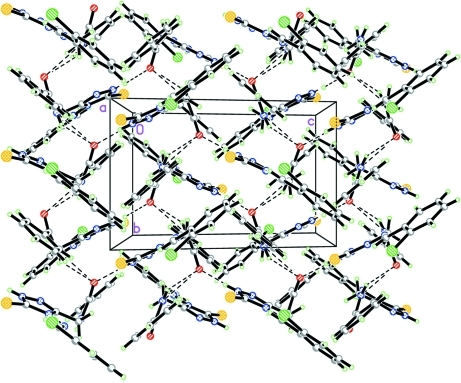
Projection down the a axis onto one two-dimensional hydrogen-bond network.

## supplementary crystallographic information

### Comment

1,2,4-Triazoles and their derivatives represent a rapidly developing field in modern heterocyclic chemistry, in part due to their antibacterial, antifungal, antitubercular, anticancer (Kalluraya *et al.*, 1996), antitumor (Al-Soud *et al.*, 2003), anticonvulsant (Almasirad *et al.*, 2004), anti-inflammatory, and analgesic properties (Amir & Shikha, 2004). Certain 1,2,4-triazoles also find applications in the preparation of photographic plates, polymers, and as analytical agents (Kawashima *et al.*, 1986). In continuation of our interest in the synthesis of chemically and biologically important heterocycles, we report here a substituted 1,2,4-triazole Schiff base.

In the title compound (Fig. 1), the dihedral angles formed by the triazole (N2/N3/C10/N4/C9) ring with the two benzene rings (C1–C6; C12–C17) are 66.88 (3)° and 19.16 (3)° respectively. The benzene rings (C1–C6; C12–C17) form a dihedral angle of 78.40 (3)°, indicating that they are inclined to each other. The structure contains intermolecular N—H···O hydrogen bonds (see Table), linking the molecules into two-dimensional networks parallel to the (100) planes (Fig. 2). Between layers, π—π stacking interactions are formed between inversion-related benzene rings (C12–C17 and its symmetry equivalent 2-*x*, 2-*y*, 1-*z*) with centroid-centroid distance 3.7717 (5) Å.

### Experimental

The title compound was obtained by refluxing *N*-[(4-amino-5-sulfanyl-4*H*-1, 2,4-triazol-3-yl)methyl]benzamide (0.01 mol) and 2-chlorobenzaldehyde (0.01 mol) in ethanol (30 ml) with 3 drops of concentrated sulfuric acid for 5 h. The solid product obtained was collected by filtration, washed with ethanol and dried. The product was then recrystallized using ethanol.

### Refinement

The amino H atoms were located in a difference map and refined with restraints of N—H = 0.85 (1) Å. The remaining H atoms were positioned geometrically [C—H = 0.93Å (aromatic) or 0.97Å (methylene)] and refined using a riding model, with *U*_iso_(H) = 1.2 *U*_eq_(C).

### Crystal data

**Table:**

C_17_H_14_ClN_5_OS	*F*(000) = 768
*M**_r_* = 371.84	*D*_x_ = 1.454 Mg m^−^^3^
Monoclinic, *P*2_1_/*c*	Mo *K*α radiation, λ = 0.71073 Å
Hall symbol: -P 2ybc	Cell parameters from 9004 reflections
*a* = 17.0185 (6) Å	θ = 2.6–26.3°
*b* = 8.0905 (3) Å	µ = 0.36 mm^−^^1^
*c* = 12.8292 (5) Å	*T* = 100 K
β = 105.962 (2)°	Plate, colourless
*V* = 1698.32 (11) Å^3^	0.70 × 0.48 × 0.15 mm
*Z* = 4	

### Data collection

**Table:**

Bruker SMART APEXII CCD diffractometer	7463 independent reflections
Radiation source: fine-focus sealed tube	6349 reflections with *I* > 2σ(*I*)
graphite	*R*_int_ = 0.025
φ and ω scans	θ_max_ = 35.0°, θ_min_ = 1.2°
Absorption correction: multi-scan (*SADABS*; Bruker, 2005)	*h* = −27→27
*T*_min_ = 0.785, *T*_max_ = 0.949	*k* = −12→13
57891 measured reflections	*l* = −20→20

### Refinement

**Table:**

Refinement on *F*^2^	Primary atom site location: structure-invariant direct methods
Least-squares matrix: full	Secondary atom site location: difference Fourier map
*R*[*F*^2^ > 2σ(*F*^2^)] = 0.033	Hydrogen site location: inferred from neighbouring sites
*wR*(*F*^2^) = 0.098	H atoms treated by a mixture of independent and constrained refinement
*S* = 1.09	*w* = 1/[σ^2^(*F*_o_^2^) + (0.0502*P*)^2^ + 0.4785*P*] where *P* = (*F*_o_^2^ + 2*F*_c_^2^)/3
7463 reflections	(Δ/σ)_max_ = 0.004
234 parameters	Δρ_max_ = 0.59 e Å^−^^3^
2 restraints	Δρ_min_ = −0.23 e Å^−^^3^

### Special details

**Table:**

Geometry. All e.s.d.'s (except the e.s.d. in the dihedral angle between two l.s. planes) are estimated using the full covariance matrix. The cell e.s.d.'s are taken into account individually in the estimation of e.s.d.'s in distances, angles and torsion angles; correlations between e.s.d.'s in cell parameters are only used when they are defined by crystal symmetry. An approximate (isotropic) treatment of cell e.s.d.'s is used for estimating e.s.d.'s involving l.s. planes.
Refinement. Refinement of *F*^2^ against ALL reflections. The weighted *R*-factor *wR* and goodness of fit *S* are based on *F*^2^, conventional *R*-factors *R* are based on *F*, with *F* set to zero for negative *F*^2^. The threshold expression of *F*^2^ > σ(*F*^2^) is used only for calculating *R*-factors(gt) *etc*. and is not relevant to the choice of reflections for refinement. *R*-factors based on *F*^2^ are statistically about twice as large as those based on *F*, and *R*- factors based on ALL data will be even larger.

### Fractional atomic coordinates and isotropic or equivalent isotropic displacement parameters (Å^2^)

**Table:**

	*x*	*y*	*z*	*U*_iso_*/*U*_eq_	
Cl1	1.007210 (13)	1.03657 (3)	0.261198 (17)	0.02066 (5)	
S1	0.757666 (14)	1.12198 (3)	0.021681 (19)	0.02205 (6)	
O1	0.52774 (4)	1.20878 (8)	0.36983 (5)	0.01823 (12)	
N1	0.52114 (5)	0.94734 (9)	0.30979 (6)	0.01683 (13)	
N2	0.56478 (5)	1.09220 (10)	0.13014 (6)	0.01817 (13)	
N3	0.60840 (5)	1.13484 (10)	0.05794 (6)	0.01859 (14)	
N4	0.69550 (4)	1.02508 (9)	0.19194 (6)	0.01486 (12)	
N5	0.75857 (4)	0.95136 (10)	0.26923 (6)	0.01636 (13)	
C1	0.37543 (6)	1.16562 (13)	0.41825 (8)	0.02102 (16)	
H1A	0.4129	1.2333	0.4659	0.025*	
C2	0.29456 (7)	1.16030 (15)	0.42187 (9)	0.0288 (2)	
H2A	0.2779	1.2232	0.4726	0.035*	
C3	0.23860 (7)	1.06069 (16)	0.34943 (11)	0.0332 (3)	
H3A	0.1846	1.0569	0.3522	0.040*	
C4	0.26273 (6)	0.96688 (15)	0.27305 (10)	0.0308 (2)	
H4A	0.2247	0.9019	0.2241	0.037*	
C5	0.34392 (6)	0.96984 (12)	0.26950 (8)	0.02199 (17)	
H5A	0.3603	0.9064	0.2188	0.026*	
C6	0.40030 (5)	1.06870 (11)	0.34268 (7)	0.01574 (14)	
C7	0.48744 (5)	1.07999 (10)	0.34185 (6)	0.01337 (13)	
C8	0.60617 (5)	0.94817 (11)	0.30987 (7)	0.01689 (14)	
H8A	0.6263	0.8354	0.3162	0.020*	
H8B	0.6376	1.0090	0.3727	0.020*	
C9	0.61919 (5)	1.02410 (10)	0.20985 (7)	0.01548 (14)	
C10	0.68828 (5)	1.09511 (11)	0.09059 (7)	0.01650 (14)	
C11	0.83182 (5)	0.96240 (11)	0.26128 (7)	0.01659 (14)	
H11A	0.8436	1.0204	0.2048	0.020*	
C12	0.89642 (5)	0.87989 (10)	0.34477 (6)	0.01453 (13)	
C13	0.97907 (5)	0.90723 (10)	0.35310 (6)	0.01472 (13)	
C14	1.04079 (5)	0.83357 (11)	0.43414 (7)	0.01758 (15)	
H14A	1.0954	0.8540	0.4387	0.021*	
C15	1.01981 (6)	0.72926 (11)	0.50811 (7)	0.01963 (16)	
H15A	1.0606	0.6804	0.5630	0.024*	
C16	0.93781 (6)	0.69731 (12)	0.50042 (7)	0.02018 (16)	
H16A	0.9240	0.6264	0.5497	0.024*	
C17	0.87707 (5)	0.77125 (11)	0.41949 (7)	0.01766 (14)	
H17A	0.8226	0.7487	0.4145	0.021*	
H1N3	0.5848 (9)	1.1788 (19)	−0.0026 (9)	0.034 (4)*	
H1N1	0.4932 (9)	0.8627 (15)	0.2855 (13)	0.040 (4)*	

### Atomic displacement parameters (Å^2^)

**Table:**

	*U*^11^	*U*^22^	*U*^33^	*U*^12^	*U*^13^	*U*^23^
Cl1	0.01679 (9)	0.02638 (11)	0.01858 (9)	−0.00361 (7)	0.00446 (7)	0.00349 (7)
S1	0.01723 (10)	0.03144 (12)	0.01831 (10)	0.00069 (8)	0.00629 (7)	0.00525 (8)
O1	0.0181 (3)	0.0162 (3)	0.0190 (3)	−0.0035 (2)	0.0027 (2)	−0.0028 (2)
N1	0.0142 (3)	0.0155 (3)	0.0217 (3)	−0.0019 (2)	0.0065 (2)	−0.0035 (2)
N2	0.0139 (3)	0.0220 (3)	0.0183 (3)	0.0027 (3)	0.0040 (2)	0.0024 (3)
N3	0.0147 (3)	0.0234 (3)	0.0168 (3)	0.0032 (3)	0.0030 (2)	0.0044 (3)
N4	0.0115 (3)	0.0184 (3)	0.0143 (3)	0.0024 (2)	0.0029 (2)	0.0018 (2)
N5	0.0130 (3)	0.0202 (3)	0.0147 (3)	0.0035 (2)	0.0020 (2)	0.0013 (2)
C1	0.0211 (4)	0.0247 (4)	0.0198 (4)	0.0061 (3)	0.0098 (3)	0.0049 (3)
C2	0.0242 (5)	0.0374 (5)	0.0302 (5)	0.0127 (4)	0.0163 (4)	0.0145 (4)
C3	0.0160 (4)	0.0428 (6)	0.0430 (6)	0.0060 (4)	0.0117 (4)	0.0218 (5)
C4	0.0146 (4)	0.0349 (6)	0.0396 (6)	−0.0037 (4)	0.0015 (4)	0.0115 (4)
C5	0.0158 (4)	0.0223 (4)	0.0255 (4)	−0.0022 (3)	0.0017 (3)	0.0029 (3)
C6	0.0135 (3)	0.0171 (3)	0.0170 (3)	0.0011 (3)	0.0049 (3)	0.0037 (3)
C7	0.0137 (3)	0.0147 (3)	0.0115 (3)	−0.0003 (2)	0.0032 (2)	0.0006 (2)
C8	0.0138 (3)	0.0203 (4)	0.0173 (3)	0.0020 (3)	0.0055 (3)	0.0012 (3)
C9	0.0125 (3)	0.0175 (3)	0.0165 (3)	0.0018 (3)	0.0040 (3)	0.0000 (3)
C10	0.0148 (3)	0.0184 (3)	0.0155 (3)	0.0009 (3)	0.0029 (3)	0.0014 (3)
C11	0.0134 (3)	0.0196 (4)	0.0160 (3)	0.0014 (3)	0.0028 (3)	0.0018 (3)
C12	0.0126 (3)	0.0161 (3)	0.0143 (3)	0.0014 (2)	0.0027 (2)	−0.0004 (2)
C13	0.0135 (3)	0.0161 (3)	0.0143 (3)	0.0009 (3)	0.0032 (2)	−0.0006 (2)
C14	0.0135 (3)	0.0193 (4)	0.0180 (3)	0.0028 (3)	0.0011 (3)	−0.0015 (3)
C15	0.0192 (4)	0.0188 (4)	0.0184 (3)	0.0046 (3)	0.0008 (3)	0.0018 (3)
C16	0.0218 (4)	0.0196 (4)	0.0187 (4)	0.0020 (3)	0.0048 (3)	0.0038 (3)
C17	0.0160 (3)	0.0196 (4)	0.0177 (3)	0.0008 (3)	0.0051 (3)	0.0016 (3)

### Geometric parameters (Å, °)

**Table:**

Cl1—C13	1.7393 (9)	C4—C5	1.3951 (14)
S1—C10	1.6726 (9)	C4—H4A	0.930
O1—C7	1.2449 (10)	C5—C6	1.3951 (13)
N1—C7	1.3340 (11)	C5—H5A	0.930
N1—C8	1.4468 (11)	C6—C7	1.4887 (11)
N1—H1N1	0.84 (1)	C8—C9	1.4930 (12)
N2—C9	1.2984 (11)	C8—H8A	0.970
N2—N3	1.3807 (11)	C8—H8B	0.970
N3—C10	1.3467 (11)	C11—C12	1.4677 (12)
N3—H1N3	0.85 (1)	C11—H11A	0.930
N4—C9	1.3797 (11)	C12—C13	1.3987 (11)
N4—N5	1.3804 (10)	C12—C17	1.4047 (12)
N4—C10	1.3924 (11)	C13—C14	1.3924 (12)
N5—C11	1.2816 (11)	C14—C15	1.3879 (13)
C1—C2	1.3905 (14)	C14—H14A	0.930
C1—C6	1.3999 (13)	C15—C16	1.3958 (13)
C1—H1A	0.930	C15—H15A	0.930
C2—C3	1.3904 (19)	C16—C17	1.3836 (12)
C2—H2A	0.930	C16—H16A	0.930
C3—C4	1.3879 (19)	C17—H17A	0.930
C3—H3A	0.930		
			
C7—N1—C8	120.72 (7)	N1—C8—H8A	109.0
C7—N1—H1N1	121.3 (12)	C9—C8—H8A	109.0
C8—N1—H1N1	117.8 (12)	N1—C8—H8B	109.0
C9—N2—N3	103.64 (7)	C9—C8—H8B	109.0
C10—N3—N2	114.49 (7)	H8A—C8—H8B	107.8
C10—N3—H1N3	124.6 (11)	N2—C9—N4	111.50 (7)
N2—N3—H1N3	120.8 (11)	N2—C9—C8	127.53 (8)
C9—N4—N5	117.35 (7)	N4—C9—C8	120.96 (7)
C9—N4—C10	108.21 (7)	N3—C10—N4	102.12 (7)
N5—N4—C10	134.27 (7)	N3—C10—S1	127.13 (7)
C11—N5—N4	119.60 (7)	N4—C10—S1	130.74 (7)
C2—C1—C6	119.76 (10)	N5—C11—C12	117.39 (8)
C2—C1—H1A	120.1	N5—C11—H11A	121.3
C6—C1—H1A	120.1	C12—C11—H11A	121.3
C3—C2—C1	119.87 (10)	C13—C12—C17	117.80 (7)
C3—C2—H2A	120.1	C13—C12—C11	121.30 (7)
C1—C2—H2A	120.1	C17—C12—C11	120.90 (8)
C4—C3—C2	120.50 (10)	C14—C13—C12	121.71 (8)
C4—C3—H3A	119.7	C14—C13—Cl1	118.16 (6)
C2—C3—H3A	119.7	C12—C13—Cl1	120.13 (6)
C3—C4—C5	120.11 (11)	C15—C14—C13	119.19 (8)
C3—C4—H4A	119.9	C15—C14—H14A	120.4
C5—C4—H4A	119.9	C13—C14—H14A	120.4
C6—C5—C4	119.47 (10)	C14—C15—C16	120.29 (8)
C6—C5—H5A	120.3	C14—C15—H15A	119.9
C4—C5—H5A	120.3	C16—C15—H15A	119.9
C5—C6—C1	120.28 (8)	C17—C16—C15	119.94 (8)
C5—C6—C7	122.17 (8)	C17—C16—H16A	120.0
C1—C6—C7	117.52 (8)	C15—C16—H16A	120.0
O1—C7—N1	120.84 (8)	C16—C17—C12	121.04 (8)
O1—C7—C6	121.35 (8)	C16—C17—H17A	119.5
N1—C7—C6	117.81 (7)	C12—C17—H17A	119.5
N1—C8—C9	112.71 (7)		
			
C9—N2—N3—C10	0.01 (11)	C10—N4—C9—C8	176.80 (8)
C9—N4—N5—C11	−173.61 (8)	N1—C8—C9—N2	3.33 (13)
C10—N4—N5—C11	11.79 (14)	N1—C8—C9—N4	−175.30 (7)
C6—C1—C2—C3	−0.94 (15)	N2—N3—C10—N4	−1.18 (10)
C1—C2—C3—C4	−0.30 (16)	N2—N3—C10—S1	177.48 (7)
C2—C3—C4—C5	1.02 (16)	C9—N4—C10—N3	1.84 (9)
C3—C4—C5—C6	−0.50 (15)	N5—N4—C10—N3	176.80 (9)
C4—C5—C6—C1	−0.73 (14)	C9—N4—C10—S1	−176.74 (7)
C4—C5—C6—C7	−178.80 (8)	N5—N4—C10—S1	−1.79 (15)
C2—C1—C6—C5	1.45 (13)	N4—N5—C11—C12	−179.19 (7)
C2—C1—C6—C7	179.61 (8)	N5—C11—C12—C13	−169.43 (8)
C8—N1—C7—O1	1.88 (12)	N5—C11—C12—C17	10.21 (12)
C8—N1—C7—C6	−178.27 (7)	C17—C12—C13—C14	−1.71 (12)
C5—C6—C7—O1	148.96 (9)	C11—C12—C13—C14	177.95 (8)
C1—C6—C7—O1	−29.16 (12)	C17—C12—C13—Cl1	178.51 (6)
C5—C6—C7—N1	−30.88 (12)	C11—C12—C13—Cl1	−1.83 (11)
C1—C6—C7—N1	151.00 (8)	C12—C13—C14—C15	0.51 (13)
C7—N1—C8—C9	−83.83 (10)	Cl1—C13—C14—C15	−179.70 (7)
N3—N2—C9—N4	1.23 (10)	C13—C14—C15—C16	0.73 (13)
N3—N2—C9—C8	−177.50 (8)	C14—C15—C16—C17	−0.71 (14)
N5—N4—C9—N2	−177.97 (7)	C15—C16—C17—C12	−0.54 (14)
C10—N4—C9—N2	−2.04 (10)	C13—C12—C17—C16	1.72 (13)
N5—N4—C9—C8	0.86 (12)	C11—C12—C17—C16	−177.94 (8)

### Hydrogen-bond geometry (Å, °)

**Table:**

*D*—H···*A*	*D*—H	H···*A*	*D*···*A*	*D*—H···*A*
N3—H1N3···O1^i^	0.85 (1)	1.89 (1)	2.7362 (10)	175 (1)
N1—H1N1···O1^ii^	0.84 (1)	2.29 (1)	2.9450 (10)	134 (2)

Symmetry codes: (i) *x*, −*y*+5/2, *z*−1/2; (ii) −*x*+1, *y*−1/2, −*z*+1/2.
